# HMGA2 promotes glioma invasion and poor prognosis via a long‐range chromatin interaction

**DOI:** 10.1002/cam4.1534

**Published:** 2018-05-07

**Authors:** Shanshan Zhang, Huibian Zhang, Lin Yu

**Affiliations:** ^1^ Department of Radiology Tianjin Medical University General Hospital Tianjin China; ^2^ Department of Biochemistry and Molecular Biology School of Basic Medical Sciences Tianjin Medical University Tianjin China

**Keywords:** glioma, HMGA2, invasion, long‐range chromatin interaction, prognostic biomarker

## Abstract

To identify the function and underlying mechanisms of HMGA2 on the prognosis and invasion of gliomas, HMGA2 was detected by immunohistochemistry. The Kaplan‐Meier and Cox's regression analysis results showed that higher HMGA2 level predicted the poorer outcomes of glioma patients. ChIP‐qPCR, DNA electrophoretic mobility shift assay, chromosome conformation capture, and co‐immunoprecipitation were applied to identify HMGA2‐activated target sites, which were further verified by mRNA and protein expression detection. Transwell and orthotopic implantation were used to investigate the roles of HMGA2 in glioma cells. HMGA2 shRNA transfection inhibited glioblastoma invasion. Mechanistically, we first discovered that HMGA2, together with GCN5, facilitated the invasion of glioma cells via inducing chromatin conformational remodeling of the *MMP2* gene promoter and epigenetically activating *MMP2* gene transcription. Our results indicated that HMGA2, as a novel GCN5 recognition partner and histone acetylation modulator, may be novel prognostic indicator and promising glioma treatment target.

## 1 INTRODUCTION

Gliomas have the highest incidence among primary brain tumors.^1^, ^2^ Among high‐grade glioma, glioblastoma (GBM) exhibits especially lethal and poor prognosis.^3^, ^4^ Significant intragroup variations in the prognosis among glioma patients interfere with the clinical diagnosis.^5^, ^6^, ^7^ The current glioma histopathologic diagnosis criteria, which are widely used, are unable to comprehensively evaluate the patients’ status and estimate their survival.^8^, ^9^, ^10^ Based on our understanding of the molecular and genetic changes in gliomas, we explore new diagnostic markers, prognostic evaluation factors, and therapy approaches for gliomas.

High‐mobility group AT‐hook 2 (HMGA2) is a member of high‐mobility group protein family. There are three independent AT‐hook domains in the C‐terminal of HMGA2, acting as DNA‐binding domains.^11^, ^12^, ^13^ By interacting with the AT‐rich sequence of DNA minor grooves, HMGA2 induces an alteration in chromatin architecture and regulates the assembling and maintenance of enhancer complexes.^14^ By controlling the expression of a series of tumor‐related genes, HMGA2 may play an important role in oncogenesis processes.^15^, ^16^ HMGA2 was shown to promote proliferation, invasion, migration, and poor prognosis in different cancers.^17^, ^18^, ^19^ However, the molecular mechanisms by which HMGA2 regulates the malignant phenotype in GBM are unclear. Our results first discovered that HMGA2 acted as a new partner of histone acetyltransferase (HAT) GCN5 and recruited it to specific positions. The HMGA2/GCN5 complex bound the AT‐rich region of DNA and catalyzed histone acetylation of the adjacent position. HMGA2 also induced a chromatin conformational change in the promoter region of matrix metalloproteinase 2 (MMP2), a well‐documented extracellular matrix regulator^20^, ^21^ and invasion factor.^21^, ^22^, ^23^ HMGA2 recruited the enhancer complex to transcription start site (TSS) of MMP2 and promoted the gene expression and invasion phenotype of GBM cells. Moreover, we identified HMGA2 could predict poorer prognosis of gliomas independent of other factors, such as IDH mutation. MMP2, not as precise as HMGA2, could still auxiliarily indicate glioma poor prognosis. Our findings suggested that HMGA2 and MMP2 may be promising prognostic markers and potential therapy site in malignant gliomas.

## 2 MATERIALS AND METHODS

### 2.1 Clinical data and tissue samples

Here, 147 cases of astrocytic gliomas were surgically collected as specimens, and 20 cases of nonmalignant brain samples were used as control. The samples were provided by Tianjin Medical University General Hospital (TMUGH), and all patients had been informed and had written consent. The formaldehyde‐fixed, paraffin‐embedded (FFPE) tissue samples were cut into 5‐μm tissue sections for HE and immunohistochemical staining. Two independent neuropathologists gave the histopathologic diagnoses based on the central nervous system tumor classification criteria from the World Health Organization (WHO). The tumor classifications, the clinical information, and WHO subclassification of patients are summarized in Table S1. This study was conducted under the guidance of the Helsinki Declaration. The Ethics Committee of TMUGH had validated all the methods in the manuscript.

The prognoses and mRNA data of 479 GBM patients from the TCGA dataset were analyzed to identify HMGA2 downstream target genes in gliomas (https://cancergenome.nih.gov/). The mRNA data from 479 GBM patients were applied to validate the correlations and the prognostic values of HMGA2 and MMP2.

### 2.2 Immunohistochemistry (IHC)

HMGA2 and MMP2 were detected by the VECTASTAIN ABC Detection System (VECTOR, Burlingame, USA) following the manufacturer's instructions. Primary antibodies included mouse anti‐HMGA2 diluted at 1:400 (Abcam, Cambridge, USA) and rabbit anti‐MMP2 diluted at 1:100 (CST, Danvers, USA). The IHC staining was analyzed with a Leica DM6000B upright microscope with CCK digital camera (Wetzlar, Germany). The labeling index [LI (%)] was calculated based on the percentage of cell number between positive staining and total.

### 2.3 Cell culture

U87MG cells were obtained from the American Type Culture Collection (ATCC, Manassas, USA) in July 2017. U251 cells were obtained from China Center for Type Culture Collection (CCTCC, Shanghai, China) September 2017. DMEM (Gibco, Carlsbad, USA) containing 10% FBS (Gibco) was used to culture the cells. All cells were incubated at 37°C with 5% CO_2_, and only the cells cultured less 15 passages were used for experiments. MycoProbe Mycoplasma Detection Kit was used to detect mycoplasma contamination (R&D, Minneapolis, USA), and the latest test was performed on 23 November 2017.

### 2.4 Migration and invasion assays

Migration and invasion experiments were performed as previously described.^24^, ^25^ U87MG and U251 cells (3 × 10^4^ cells/well) were subjected to the upper level of a transwell insert (Millipore, Billerica, USA) with or without matrigel (BD Bioscience, USA). After 24 hours, the membranes were stained with crystal violet and the positive staining cells were randomly counted under a Leica DM6000B upright microscope (Wetzlar, Germany, nine fields, ×400).

### 2.5 ChIP‐qPCR assays

EZ‐Magna ChIP^™^ Kit (Millipore, Billerica, USA) was used for ChIP‐qPCR assay. The anti‐HMGA2 antibody (8 μg; Abcam, Cambridge, USA) and anti‐IgG antibody (8 μg; Sigma, St. Louis, USA) were used to precipitate the chromatin. The ChIP was performed under the guidance of the manufacturer's instruction. The ChIP‐qPCR primers are listed in Table S2 (Sangon, Shanghai, China).

### 2.6 IDH mutation assays

We used the QIAamp DNA FFPE Tissue Kit (Qiagen, Valencia, CA) to purify the genomic DNA from the FFPE tissues. The genomic DNA concentration was then measured by a NanoDrop spectrophotometer (Thermo, Houston, TX). The IDH mutations were sequenced by Gene Tech (Shanghai, China).

### 2.7 Orthotopic implantation of GBM cell lines

U87MG cells were first transfected with a lentivirus expressing firefly luciferase (Xenogen). The cells were then further infected with lentiviruses bearing an empty control (Con) or full‐length HMGA2‐coding sequence (HMGA2). The cells were also cotransfected with scrambled shRNA plus an empty vector (Scramble/Con) or scrambled shRNA or GCN5 shRNA plus the HMGA2 ectopic expression vector (Scramble/HMGA2, GCN5‐sh1/HMGA2, GCN5‐sh2/HMGA2). The Table S3 contains the shRNA sequences we used. These cells were implanted (7.5 × 10^4^ cells) into the brains of 6‐week‐old NOD‐SCID mice. After 28 days, d‐luciferin was injected to mice in 200 mg/g, and the bioluminescence was imaged and quantified by IVIS system (Xenogen, Waltham, USA). Animal handling and procedures followed the principles of laboratory animal care of NIH and were approved by the Tianjin Medical University Institutional Animal Care and Use Committee.

### 2.8 DNA electrophoretic mobility shift assay (EMSA)

The biotin‐labeled, unlabeled, and mutated probes of the MMP2 promoter were purchased from Beyotime (Shanghai, China; Table S4). We used the LightShift Chemiluminescent EMSA Kit (Thermo, Rockford, USA) to do the EMSA assay. The reaction mixtures included a negative control mixture containing 20 fmol biotin‐labeled probe, a binding reaction with 10 μg nuclear extract and 20 fmol biotin‐labeled probe, unlabeled probe competition reaction gradient mixtures containing 10 μg nuclear extract, 20 fmol biotin‐labeled probe, and unlabeled probe from 100 to 1000 fmol. After a 30‐minute incubation at room temperature, the samples were subjected to 7% native PAGE gel electrophoresis (85 V, 45 minutes).

### 2.9 RNA extraction and mRNA qRT‐PCR

TRIzol reagent was used to extract total RNA (Invitrogen, Carlsbad, USA). The cDNA was generated by RevertAid First Strand cDNA Synthesis Kit (Fermentas, Pittsburgh, USA). The qPCR reactions were performed with FastStart Universal SYBR Green Master Mix (Roche, Nutley, USA) on a StepOne^™^ Real‐Time PCR System (Applied Biosystems, USA, 95°C, 30 seconds for denaturation, 60°C, 1 minute for annealing and extension, 40 cycles). The relative mRNA level was qualified by ΔΔ*C*_*T*_ method, and GAPDH was selected as an internal reference. Primer sequences are listed in Table S5 (Sangon, Shanghai, China).

### 2.10 Western blot assay

Proteins were extracted by enhanced RIPA buffer with phenylmethylsulfonyl fluoride (PMSF, Sigma, St. Louis, USA) and EDTA. The protein concentrations were quantified with the BCA method. The total protein (20 μg) of each sample was loaded to SDS‐PAGE and detected with the appropriate antibodies.

### 2.11 Chromosome conformation capture

Cells (1 × 10^6^) were treated with formaldehyde to fix the chromatin conformation; the chromatin was isolated and treated with NlaIII for fragmentation. Then, we treat the chromatin fragments with high‐concentration T4 DNA ligase to induce cross‐linking self‐ligation. The cross‐linking ligation efficiencies were further detected by PCR. A PCR product from 5 kb upstream of the MMP2 promoter was randomly digested and ligated by NlaIII, which served to generate standards. The ligation efficiencies among the different samples and different experiments were normalized by the ligation efficiency of each experimental series. Primer sequences are listed in Table S6.

### 2.12 Statistical analyses

SPSS 21.0 software was used to generate all the statistical analysis results (IBM, Chicago, Illinois). Data are presented as the mean ± SD. One‐way ANOVA was used as mean comparison method among different groups. The correlations among the *HMGA2* and *MMP2* mRNA (TCGA data) and protein (our data) expression were measured by Pearson's correlation. The Kaplan‐Meier method was used to analyze patient survival time (disease‐free survival: DFS, overall survival: OS). The medians of the corresponding gene levels were used to stratify subgroups in all survival analyses. The Cox's regression was applied to analyze univariate and multivariate survival factors. *P*‐values <.05 were considered as a statistical significance; *P *<* *.05 (*), *P *<* *.01 (**), or *P *<* *.001 (***). All cell line experiments were repeated in triplicate.

## 3 RESULTS

### 3.1 HMGA2 correlates with glioma grade and informs poor patient outcome

**Figure 1:**
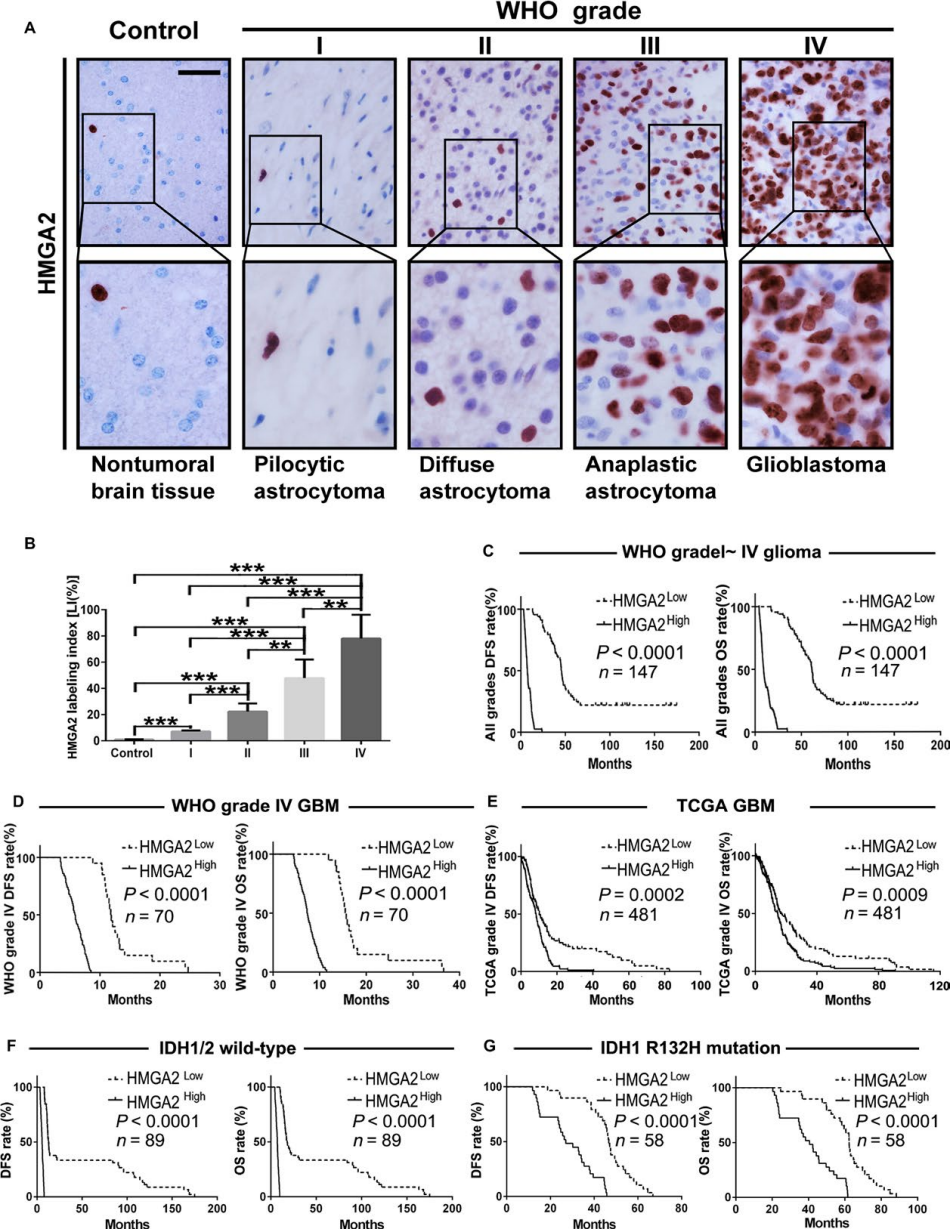
HMGA2 expression is correlated with both glioma grade and patient prognosis. A, HMGA2 IHC staining in the FFPE samples of 147 gliomas (n = 147) and 20 nonmalignant brain tissues (n = 20); scale bar = 50 μm. B, Quantification of the HMGA2 expression level (LI) among the different groups of the above brain tissue collection. The calculation of HMGA2 LI was defined in Section 2. The data are given as the mean ± SD. **, *P *<* *.01; ***, *P *<* *.001. C,D, The relationship between HMGA2 protein expression and DFS (left) or OS (right) of glioma patients of all grades (C, n = 147) and WHO grade IV GBM (D, n = 70) patients was analyzed by Kaplan‐Meier method. E, Survival test analyzed the effect of the *HMGA2* level on DFS and OS of GBM patients from the TCGA dataset, n = 481. F‐G, Survival test analyzed the effect of HMGA2 protein expression on the DFS and OS of our glioma samples with wild‐type (F, n = 89) or R132H‐mutated IDH1 (G, n = 58). The medians of the HMGA2 levels were used to stratify the low‐ and high‐expression subgroups in all of the above survival analyses

We detected HMGA2 expression in all FFPE specimens by IHC staining, including 147 glioma and 20 nontumoral brain tissue samples. The results showed that the HMGA2 level was significantly and positively correlated with glioma grade and was highest in GBM (Figure 1A,B). Kaplan‐Meier analysis further confirmed that the patients bearing high HMGA2 level had less survival time (Figure 1C). Furthermore, HMGA2 had prognostic value in GBM patients; a higher HMGA2 level in the specimens indicated a graver outcome of the original patients (Figure 1D). The outcome predictive value of HMGA2 in GBM was further verified in 481 TCGA GBM samples (Figure 1E). We also found that HMGA2 was an independent prognostic factor for glioma patient survival by multivariate and univariate analyses (Tables 1 and 2). IDH1 mutations are novel glioma diagnostic markers, and pyrosequencing results showed that 58 cases had the IDH1 R132H mutation in our 147 glioma samples. We found that the HMGA2 level was not associated with the IDH mutation condition. HMGA2 upregulation predicted shorter survival times in all glioma samples, with or without IDH mutations (Figure 1F‐G, Tables 1 and 2). These results indicate that HMGA2 is a potential IDH‐independent poor prognostic biomarker for glioma patients.

**Table 1:** Multivariate analysis for DFS and OS in patients with gliomas

Factors	DFS		OS	
	HR (95% CI)	*P*	HR (95% CI)	*P*
Gender	0.882 (0.592‐1.316)	.5401	0.835 (0.559‐1.247)	.3772
Age	1.000 (0.984‐1.016)	.9762	1.007 (0.990‐1.025)	.4133
Predominant side	0.893 (0.605‐1.320)	.5713	0.929 (0.629‐1.371)	.7092
Predominant location	1.117 (0.821‐1.274)	.1092	1.119 (0.882‐1.440)	.3014
KPS	1.031 (0.504‐2.112)	.9334	1.013 (0.485‐2.113)	.9728
WHO grade	1.239 (1.199‐1.293)	<.0001	1.402 (1.388‐1.492)	<.0001
IDH status	0.135 (0.031‐0.580)	.0068	0.498 (0.297‐0.627)	.0207
HMGA2 LI	1.467 (1.125‐1.914)	.0047	1.571 (1.460‐1.718)	.0004
MMP2 LI	1.098 (0.857‐1.407)	.4583	1.019 (0.927‐1.114)	.6188

CI, confidence interval; HR, hazard ratio; LI, labeling index.

**Table 2:** Univariate analysis for DFS and OS in patients with gliomas

Factors	DFS		OS	
	HR (95%CI)	*P*	HR (95%CI)	*P*
Gender	0.765 (0.539‐1.086)	.1314	0.758 (0.534‐1.077)	.1218
Age	1.050 (1.038‐1.061)	<.0001	1.049 (1.038‐1.061)	<.0001
Predominant side	0.787 (0.604‐1.027)	.0775	0.785 (0.603‐1.026)	.0764
Predominant location	1.809 (1.533‐2.135)	<.0001	1.800 (1.525‐2.123)	<.0001
KPS	1.102 (0.901‐1.202)	.2013	1.094 (0.874‐1.317)	.2274
WHO grade	1.309 (1.241‐1.472)	<.0001	1.340 (1.221‐1.439)	<.0001
IDH status	0.577 (0.403‐0.825)	.0025	0.572 (0.399‐0.819)	.0017
HMGA2 LI	1.394 (1.293‐1.420)	<.0001	1.294 (1.231‐1.337)	<.0001
MMP2 LI	1.132 (1.111‐1.153)	<.0001	1.130 (1.109‐1.152)	<.0001

CI, confidence interval; HR, hazard ratio; LI, labeling index.

### 3.2 HMGA2 promotes the migration and invasion phenotypes of GBM cells

**Figure 2:**
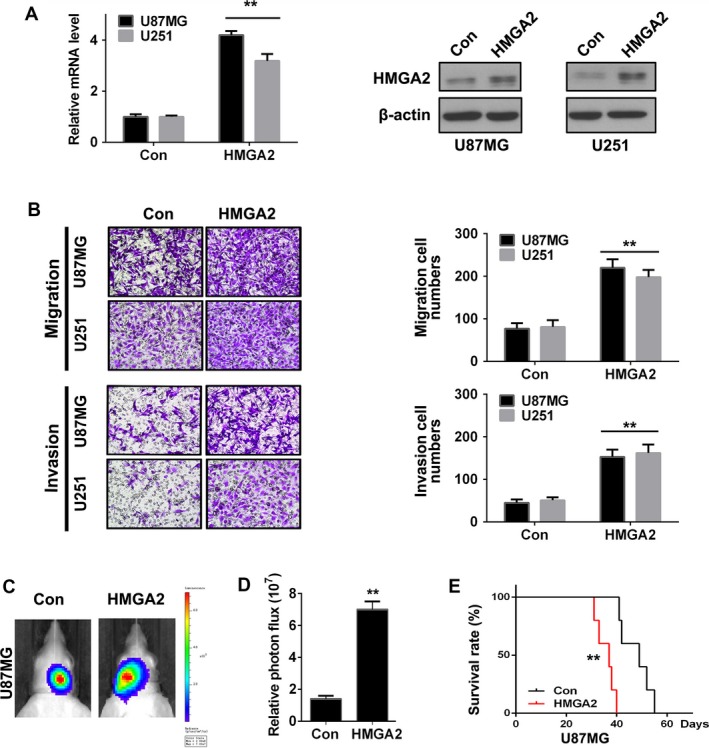
HMGA2 facilitates GBM cell invasion in vitro and malignancy in vivo. (A) qRT‐PCR and Western blotting analysis of the HMGA2 level in U87MG and U251 cells infected with control viruses (Con) or HMGA2 expression viruses (HMGA2). n = 3, **, *P *<* *.01. B, The migration and invasion of the above GBM cells were detected by transwell assay (left), and infiltrating cell numbers were qualified (right). n = 3, **, *P *<* *.01. C, Six‐week‐old NOD‐SCID mice were orthotopically inoculated with U87MG‐Con or U87MG‐HMGA2 cells. n = 5. D, Tumors were measured by IVIS imaging system at 30 days after initial implantation. n = 5, **, *P *<* *.01. E, Kaplan‐Meier analysis of the in vivo tumor transplant assay. HMGA2 group had significantly shorter survival time than Con group n = 5, **, *P *<* *.01. The data in (A, B, and D) are given in mean ± SD

GBM is lethal due to its relentless invasion and migration phenotypes. We performed transwell and orthotopic tumor implantation assays to assess the effect of HMGA2 on the GBM migration and invasion process. We found that compared with the control cells, U87MG and U251 cells with exogenous HMGA2 overexpression (HMGA2) exhibited dramatically greater migration and invasion (Figure 2A,B). Then, orthotopic tumor transplantation was further applied to confirm the oncogenic effect of HMGA2. U87MG cells were infected with a control or HMGA2 expression virus. Then, the above cells were injected into the brain of SCID mice (n = 5). The luminescence quantification results showed that the HMGA2 overexpression group had a remarkably larger tumor size than the control groups (Figure 2C,D). The HMGA2 overexpression group also had a shorter survival time, which was confirmed by Kaplan‐Meier analyses (Figure 2E). Our results revealed that HMGA2 may promote the invasion and migration phenotypes of GBM.

### 3.3 HMGA2 binds to the MMP2 gene promoter and is associated with *MMP2* promoter histone acetylation

**Figure 3:**
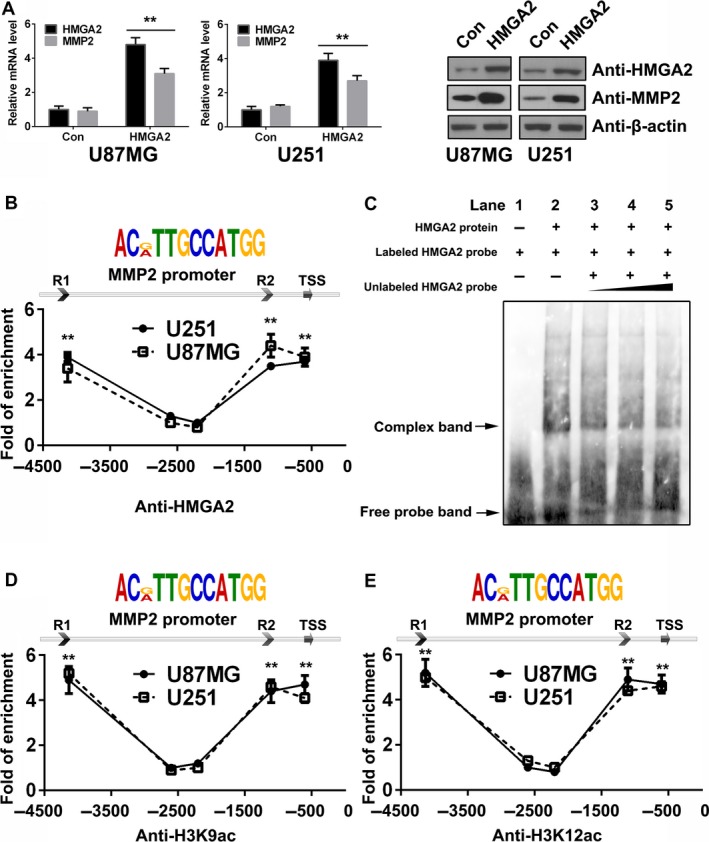
HMGA2 upregulates MMP2 transcription via binding the MMP2 prompter in GBM cells. A, mRNA and protein levels of HMGA2 and MMP2 in U87MG and U251 cells of the control (Con) and HMGA2 overexpression (HMGA2) groups. n = 3, **, *P *<* *.01. B, HMGA2 binding sites predicted in the *MMP2* promoter (top figure) and ChIP‐qPCR analyses of the capacities for them to bind with HMGA2 in U87MG and U251 cells. n = 3, **, *P *<* *.01. C, Binding between HMGA2 protein and the *MMP2* promoter verified by EMSA assay. D,E, ChIP‐qPCR was performed in U87MG and U251 cells to detect the histone H3K9ac (D) and H3K12ac (E) modification condition of the MMP2 promoter. n = 3, **, *P *<* *.01. The data in (A, B, D, and E) are given in mean ± SD

Several AT‐rich regions are located on the promoter of *MMP2*, which indicates that HMGA2 may recognize the *MMP2* promoter and regulate its transcription. We used a lentivirus with an empty vector (Con) or HMGA2 gene sequence (HMGA2) to transfect the U87MG and U251 cells. Then, the qRT‐PCR and Western blot results indicated that HMGA2 induced the amount of the MMP2 gene at both the mRNA and protein levels (Figure 3A).

To further verify whether HMGA2 could promote MMP2 transcription, we performed a ChIP‐qPCR assay. The results showed that HMGA2 was recruited to three conserved regions [R1, R2, and the transcription start site (TSS), containing an ACMTTGCCATGG sequence] of the *MMP2* promoter in GBM cells (Figure 3B). Moreover, an EMSA was performed using the HMGA2 binding sites in the *MMP2* promoter as the detecting probes. The results showed that purified HMGA2 protein generated by in vitro translation could bind to the labeled probe (complex band from the labeled HMGA2 probe, Figure 3C, Lane 2), and the unlabeled probe could competitively inhibit the formation of the complex band in a dose‐dependent manner (Figure 3C, Lanes 3 to 5). The above results demonstrated that HMGA2 protein can directly bind to the conserved DNA sequences of the *MMP2* promoter.

Histone H3K9Ac and H3K12Ac are the major histone acetylations on the enhancer and TSS. In both U87MG and U251 cells, ChIP assays targeting H3K9Ac and H3K12Ac revealed that the above two types of histone modifications were located at the same positions as HMGHA2 on the *MMP2* gene loci, indicating that HMGA2 may act as an epigenetic activation regulator of *MMP2* (Figure 3D,E). The above results revealed that HMGA2 may serve as a link in the logic chain of histone acetylation and MMP2 gene transcription.

### 3.4 HMGA2 promotes MMP2 gene transcription via an interaction with the histone acetyltransferase GCN5

**Figure 4:**
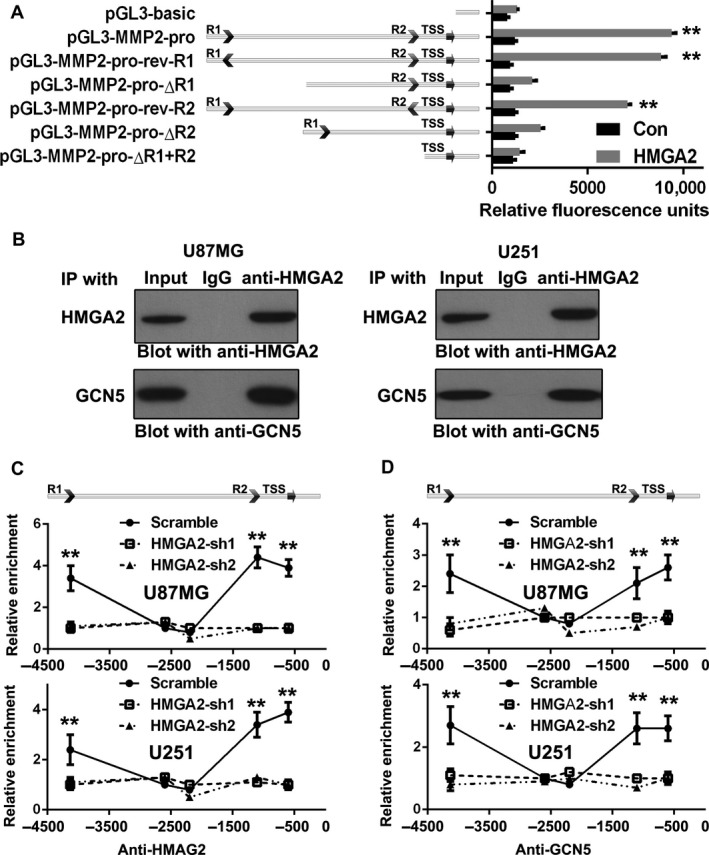
HMGA2 promotes MMP2 transcription via its interaction with GCN5. A, Luciferase reporter assays were applied with a pGL3‐basic vector containing the full‐length MMPP2 promoters or several mutations of the MMP2 promoters. The different reporter vectors were transfected along with the pRL‐CMV reference vector to U87MG with or without HMGA2 ectopic expression (Con or HMGA2). Luminescence signal was detected after 48 h of transfection. n = 3, **, *P *<* *.01. B, HMGA2 co‐IP with GCN5 in vivo; U87MG and U251 cells were immunoprecipitated with the anti‐HMGA2 antibody and blotted with antibodies against HMGA2 and GCN5. (C‐D) ChIP was performed in U87MG‐Scramble, U87MG‐HMGA2‐sh1/sh2, U251‐Scramble, and U251‐HMGA2‐sh1/sh2 cells to detect the enrichment condition of HMGA2 (C) and GCN5 (D) at the MMP2 promoter after HMGA2 knockdown. n = 3, **, *P *<* *.01. All data in (A, C, and D) are given in mean ± SD

HMGA2 is a well‐documented AT‐rich sequence‐binding protein. The HMGA2 binding sites on MMP2 loci were also modified with histone H3K9Ac and H3K12Ac, two well‐documented enhancer and TSS markers. The above results indicated that HMGA2 may bind to the enhancer or TSS of the MMP2 gene. We then tested the function of R1 and R2 as potential enhancers or the TSS of the MMP2 promoter. Luciferase reporters were transfected into both GBM cells with or without ectopic HMGA2 expression. The results showed that the −451 to +204 fragment of the whole promoter had basal transcription activity (Figure 4A, pGL3‐MMP2‐pro‐ΔR1 + R2). Serial extensions of this core promoter fragment demonstrated that inclusion of R1 + R2 but not R1 or R2 alone significantly enhanced basal promoter activity. The above results showed that in addition to the core promoter, R1 and R2 are both necessary for HMGA2 to induce MMG2 gene transcription.

On the MMP2 promoter, histones of the HMGA2 binding site were highly acetylated, while HMGA2 itself did not catalyze the acetylation process. Due to the obvious association among HMGA2 and histone acetylation, HMGA2 should recruit other histone acetyltransferases (HATs) to the MMP2 promoter. GCN5 is the most important HAT that catalyzes most of the histones on the chromatin. Previous result showed that HMGA2 could induce the expression of GCN5 in pancreatic cancer cells.^26^ In this manuscript, we identified that GCN5 is a novel HMGA2 partner which could be recruited to the *MMP2* promoter. The total cell lysates of two above GBM cells were immunoprecipitated with the anti‐HMGA2 antibody, and the normal IgG antibody served as a negative control. Then, HMGA2 and GCN5 antibodies were used for further Western blot detection. Endogenous HMGA2 was efficiently coprecipitated with endogenous GCN5 (Figure 4B, lower panel). The above results indicated the physical interaction between HMGA2 and GCN5 in glioma cells, which may play an important role in the HMGA2‐inducing histone acetylation process on *MMP2* promoters.

GCN5 requires a partner protein to distinguish the various cis‐elements on the chromatin, acetylate histones, and activate transcription. To address the essential partner function of HMGA2 in recruiting GCN5 to the MMP2 promoters in GBM cells, we performed ChIP experiments targeting HMGA2 and GCN5 in the stable U87MG and U251 cell lines with shRNA‐mediated knockdown of endogenous HMGA2 (U87MG‐HMGA2‐sh‐1, ‐sh‐2, U251‐HMGA2‐sh‐1, ‐sh‐2) and U87MG‐Scramble and U251‐Scramble cells. As expected, HMGA2 knockdown in U87MG and U251 cells dramatically inhibited the binding of HMGA2 with R1, R2, and the TSS of MMP2 promoters (Figure 4C). More interestingly, GCN5 was located at the same positions recognized by HMGA2 on MMP2 promoters in both Scramble GBM cells (R1, R2, and TSS, Figure 4D, Scramble). Knocking down of HMGA2 disturbed the GCN5 distribution on R1, R2, and the TSS region (Figure 4D, HMGA2‐sh‐1, ‐sh‐2). The above results further confirmed that HMGA2 is a key partner of GCN5 that is recruited to the *MMP2* promoter.

### 3.5 HMGA2 promotes MMP2 gene transcription via inducing histone acetylation and chromatin conformational changes

**Figure 5:**
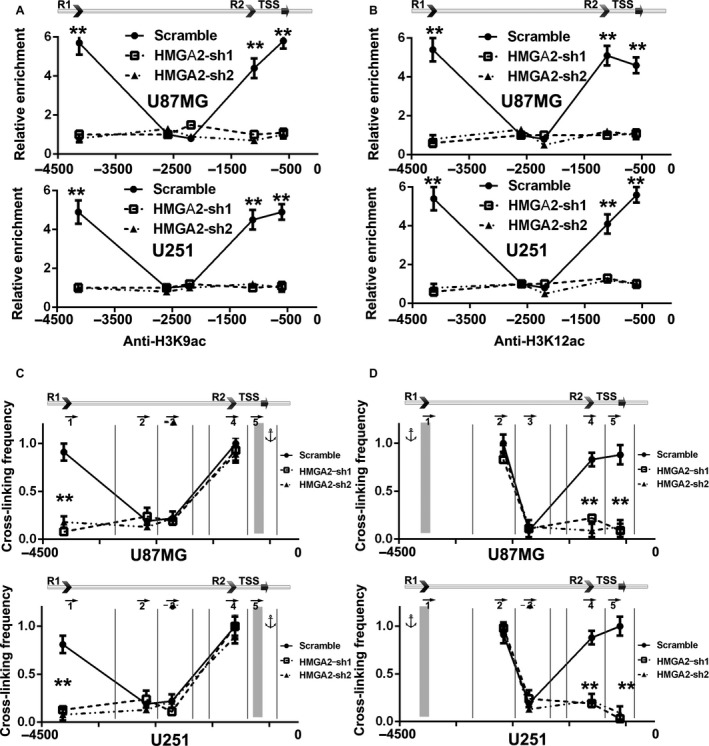
HMGA2 upregulates MMP2 transcription by inducing long‐range chromatin conformational remodeling at the MMP2 prompter. A,B, ChIP results depicted the histone H3K9 (A) and H3K12 (B) acetylation locations on the MMP2 promoter in U87MG‐Scramble, U87MG‐HMGA2‐sh1/sh2, U251‐Scramble, and U251‐HMGA2‐sh1/sh2 cells. HMGA2 knockdown inhibited the histone acetylation of the MMP2 promoter. n = 3, **, *P *<* *.01. C, 3C was performed to measure the cross‐link ligation efficiency among HMGA2 binding sites R1, R2, and the TSS in both U87MG and U251 cells with or without HMGA2 knockdown. NlaIII restriction sites of MMP2 promoter were labeled by vertical lines. The PCR primer position and direction were labeled by arrows. TSS was set as the anchor; the cross‐link efficiency between the R1 and R2 motifs is shown. n = 3, **, *P *<* *.01. (D) 3C results showed the cross‐link efficiency with the HMGA2 binding site R1 as anchor. n = 3, **, *P *<* *.01. All data in (A‐D) are given in mean ±SD

To investigate whether the recruitment of GCN5 to *MMP2* promoters would sequentially induce histone acetylation, the histone H3K9Ac and H3K12Ac modification conditions on *MMP2* promoters were detected by ChIP‐Q‐PCR. In both U87MG‐Scramble and U251‐Scramble cells, histones of the HMGA2 binding site on the *MMP2* promoter were highly acetylated, indicating epigenetic activation (Figure 5A,B). In contrast, in U87MG‐HMGA2‐sh1/2 and U251‐HMGA2‐sh1/2 cells, there were significantly fewer histone acetylations on the above HMGA2 binding site than in the scrambled controls (HMGA2‐sh‐1 and sh‐2, Figure 5A,B). The above results indicated that as a key partner in the recruitment of GCN5 to the *MMP2* promoter, HMGA2 induced histone acetylation and promoted gene transcription action of *MMP2*.

We then explored whether the conserved upstream HMGA2‐interacting R1 or R2 contained chromatin architectural function in vivo. The long‐range chromatin architectural change among cis‐regulatory elements and the TSSs of target genes requires a certain transcriptional factor and HAT to form a physical interaction with the above chromatin fragments. We used chromosome conformation capture (3C) to detect the spatial propinquity among R1, R2, and the MMP2 promoter. The purified chromatin was digested and re‐ligated with NlaIII. Then, qPCR was applied to evaluate the association frequencies among different fragments. Among each of the chromatin preparations, the cross‐link frequency was normalized by the highest value. We first set the TSS fragment of MMP as the anchor. The PCR results showed that there were very strong associations between the TSS and the R1 or R2 fragments in U87MG‐Scramble and U251‐Scramble cells (Figure 5C). As expected, HMGA2 knockdown completely inhibited the formation of the above associations (HMGA2‐sh1 and sh2, Figure 5C). Consistently, when R1 was set as the anchor, an inhibitory effect of HMGA2 knockdown on the interaction among the R1, R2, and TSS fragments was also observed (Figure 5D). Thus, in GBM cells, we found that HMGA2 induced the formation of a topological complex on the *MMP2* promoter, containing the R1, R2, and TSS fragments and the acetylated histone of the above fragments. GCN5 was recruited to R1, R2, and the TSS by HMGA2 and catalyzed the histone acetylation, which triggered the long‐range conformational change among the three loci of the *MMP2* promoter, thereby promoting *MMP2* gene transcription.

### 3.6 HMGA2 promotes MMP2 expression and GBM cell migration and invasion via GCN5

**Figure 6:**
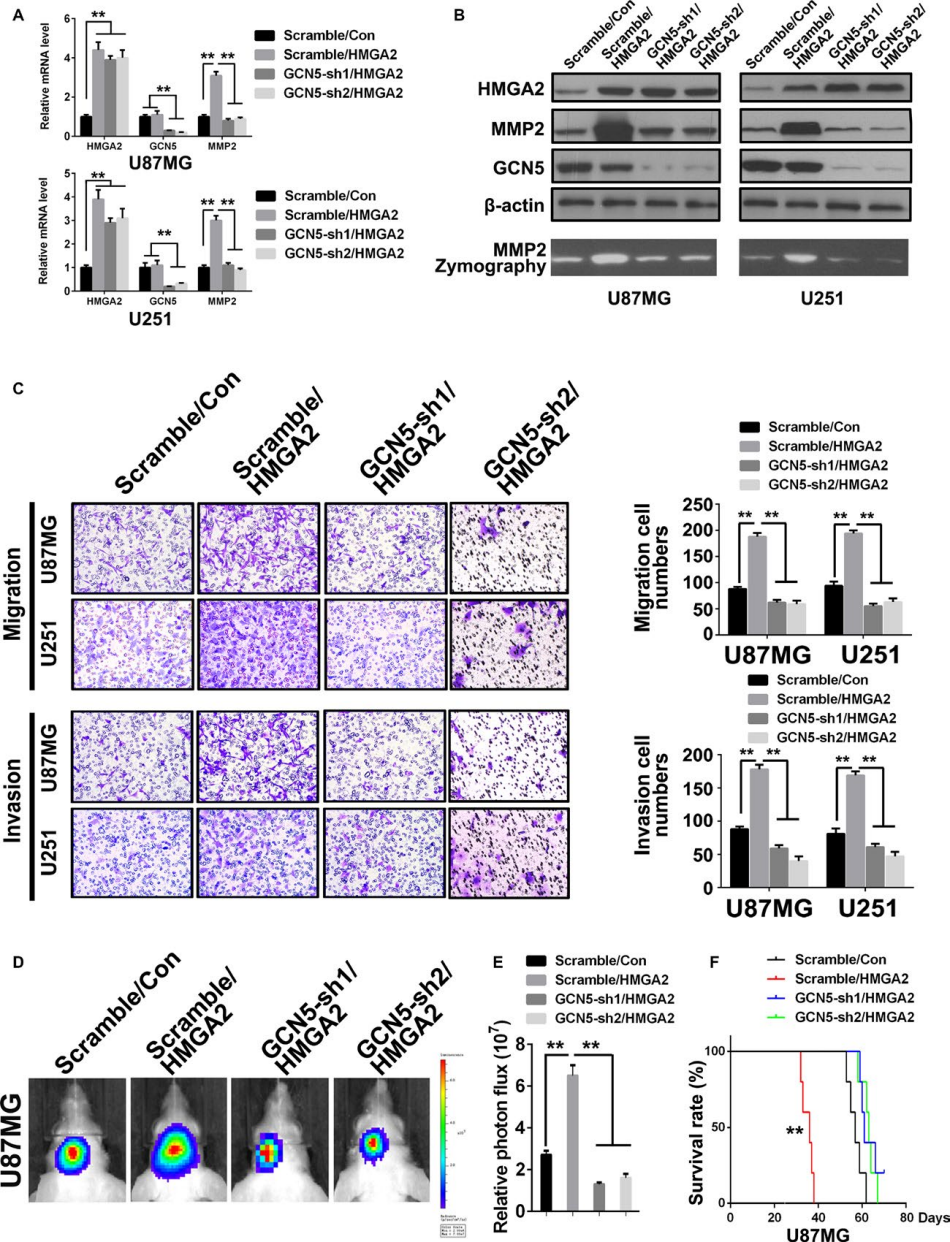
HMGA2 facilitates GBM cell malignancy via GCN5. A, qPCR analyses of the mRNA level of HMGA2, GCN5, and MMP2 in the U87MG and U251 cells of control (Scramble/Con), HMGA2 overexpression (Scramble/HMGA2), and HMGA2 overexpression with GCN5 knockdown (GCN5‐sh1/HMGA2 and GCN5‐sh2/HMGA2) groups. n = 3, **, *P *<* *.01. B, The protein levels of HMGA2, GCN5, and MMP2 in the aforementioned cells in (A). Gelatin zymography analyses of the activity of MMP2 in the aforementioned cells in (A). C, Transwell assay was performed to measure the migration and invasion ability of the aforementioned cells in (A). n = 3, **, *P *<* *.01. D, Six‐week‐old NOD‐SCID mice were orthotopically inoculated with U87MG cells infected with lentiviruses carrying an empty vector plus scramble shRNA (Scramble/Con) or ectopic HMGA2 plus scramble shRNA (Scramble/HMGA2) or GCN5 shRNA (GCN5‐sh1/HMGA2, GCN5‐sh2/HMGA2), n = 5/group. E, Tumors were quantified using IVIS luminescence image system. n = 5, **, *P *<* *.01. F, Kaplan‐Meier survival test result for in vivo tumor transplant assay. The survival in the Scramble/HMGA2 group was significantly shorter than that in other groups. n = 5, **, *P *<* *.01. The data in (A, C, E) are given in mean ± SD

To ascertain whether GCN5 mediates the promotion effect of HMGA2 on MMP2 upregulation and GBM cell migratory and invasive abilities, we constructed a lentivirus expressing GCN5 shRNAs (GCN5‐sh1, sh2). U87MG and U251 cells were transfected with scramble shRNA/vector virus (Scramble/Con; control), scramble shRNA/HMGA2 expression virus (Scramble/HMGA2; exogenous HMGA2 overexpression) or GCN5 sh‐1, sh2/HMGA2 expression virus (GCN5‐sh1, sh2/HMGA2; exogenous HMGA2 overexpression plus endogenous GCN5 knockdown). qRT‐PCR and Western demonstrated that MMP2 expression was significantly increased in Scramble/HMGA2 cells and decreased to the basal level in GCN5‐sh1/HMGA2 and GCN5‐sh2/HMGA2 cells compared with that in Scramble/Con cells (Figure 6A,B). The zymography assay showed similar results as the Western blot (Figure 6B, lower panel). Transwell assays demonstrated that the migration and invasion of Scramble/HMGA2 GBM cells were greater than those of control cells. Interestingly, GCN5 shRNA transfection effectively attenuated the above promoting effects of exogenous HMGA2 overexpression on migration and invasion (Figure 6C). The above results confirmed that GCN5 was the mediator of the HMGA2‐induced migration/invasion acceleration process in GBM cells. The in vivo transplant assay showed a similar result as the transwell assay; HMGA2 overexpression led to a marked increase in tumor size, while GCN5 knockdown neutralized the above effect (Figure 6D,E). The survival times of the different groups were analyzed by Kaplan‐Meier methods. The results showed that Scramble/HMGA2 cells had a significantly shorter survival time than the Scramble/Con group. The survival times of the GCN5 knockdown groups (GCN5‐sh1, sh2/HMGA2) were extended to the basal level and were similar to those of the control group (Scramble/Con, Figure 6F).

### 3.7 HMGA2 overexpression correlates with high levels of MMP2 in human glioma samples

**Figure 7:**
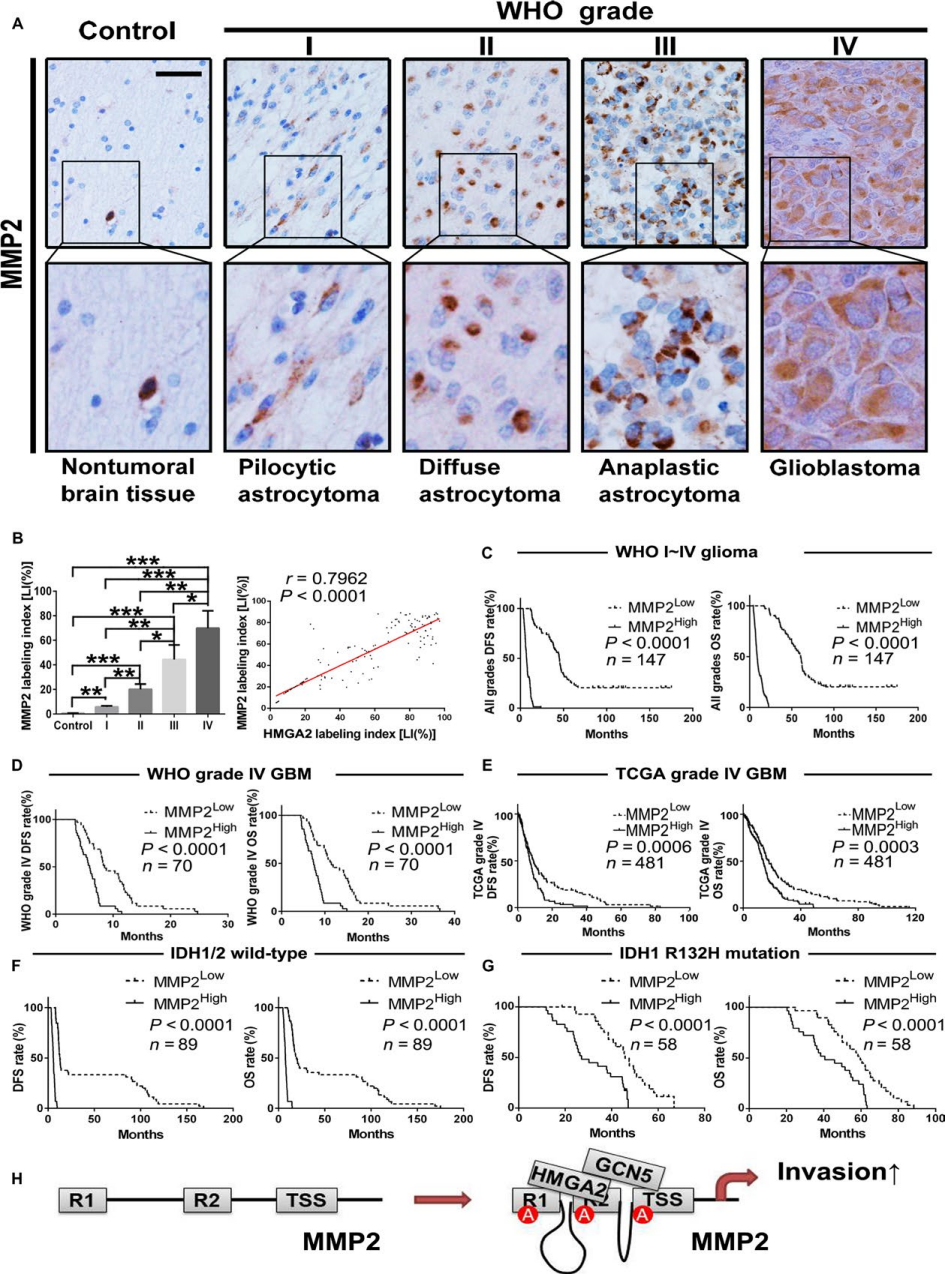
MMP2 expression correlates with glioma grades, HMGA2, and patient prognosis. A, IHC staining images of MMP2 in our brain tissue collection; scale bar = 50 μm. B, Quantification of the MMP2 LI among groups in our tissue collection. The computing method of MMP2 LIs was the same as that of HMGA2 LI and given in mean ± SD. *, *P *<* *.05; **, *P *<* *.01; ***, *P *<* *.001. Linear regression was performed to evaluate the expression correlation between HMGA2 and MMP2 in the tissue collection from 147 gliomas. C,D, Kaplan‐Meier survival curves based on MMP2 protein expression in WHO grade I‐IV glioma (C, n = 147) and grade IV GBM (D, n = 70) samples. E, Kaplan‐Meier survival curves based on *MMP2* mRNA expression in GBM patients from the TCGA database. n = 481. F‐G, Kaplan‐Meier analysis based on MMP2 level in all wild‐type IDH1/2 glioma (F, n = 89) and IDH1 R132H mutation‐bearing glioma patients (G, n = 58). The medians of the MMP2 levels were used to stratify the low‐ and high‐expression subgroups in all of the above survival analyses. H, Schematic illustration of the mechanisms by which HMGA2 promotes glioma cell invasion

To investigate whether the correlations between HMGA2 and MMP2 exist in human glioma tissues, we further detected MMP2 in our FFPE specimens by IHC and compared its expression pattern with that of HMGA2. In comparison with nontumoral brain tissues, gliomas expressed more MMP2, the expression of which significantly increased with an increase in glioma grade (Figure 7A,B). Moreover, MMP2 expression was positively correlated with HMGA2 expression in our specimens (Figure 7B, right panel), which was further verified by the open source data from the TCGA GBM dataset (Figure S1). Kaplan‐Meier analyses further confirmed that high MMP2 levels indicated a shorter survival time in our glioma (Figure 7C) and GBM (Figure 7D) patients. The above results were also verified by TCGA GBM dataset (Figure 7E). HMGA2 upregulation predicted shorter survival times in all glioma samples, with or without IDH mutations (Figure 7F‐G). Univariate analysis indicated that MMP2 could predict glioma prognosis auxiliarily (Table 2). HMGA2 serves as a recognition partner to recruit GCN5 to certain sequences of the MMP2 promoter and acetylate the adjacent histone, which finally induces a long‐range physical interaction between promoter cis‐regulating elements and transcriptional activation of MMP2. The results further enriched our understanding of the mechanism by which HMGA2 regulates the malignant phenotype of gliomas (Figure 7H).

## 4 DISCUSSION

Although HMGA2 overexpression has been detected in different malignant tissues,^27^, ^28^, ^29^, ^30^, ^31^, ^32^ the oncogenic mechanism and clinical relevance of HMGA2 in gliomas remained unclear. Our results showed increased expression of the HMGA2 protein in the majority of both GBM and WHO grade I‐III glioma tumors by IHC using a cohort of 147 grade I‐IV gliomas compared to 20 normal brain samples. Because the current histopathologic criteria for glioma diagnosis cannot accurately assess patient prognosis,^8^, ^9^, ^10^ we performed a prognosis‐based comprehensive analysis of HMGA2 in gliomas of grades to assess the diagnostic potential of HMGA2. Our results showed that the HMGA2 level was remarkably and positively correlated with glioma grade in our 147 glioma specimens, indicating that HMGA2 may act as a promising biomarker to distinguish the WHO grades of glioma. Moreover, the samples of our tumor cohort with grade I‐IV gliomas and the GBMs from the TCGA database with a higher HMGA2 level had shorter DFS and OS, and both multivariate and univariate analyses using our data identified HMGA2 as an independent predictor of glioma patient survival, indicating that HMGA2 is a specific biomarker for prognosis‐based glioma subclassification.

Malignant gliomas are characterized by high‐speed invasion and migration for tumor cells, especially GBM.^25^, ^33^, ^34^ Our results indicated that HMGA2 overexpression leads to the acceleration of cell migration and invasion in malignant gliomas, thereby expediting their progression. The multisite interactions among HMGA2 and the MMP2 promoter characterized in this study provide novel molecular insights into the mechanism by which HMGA2 promotes gene expression. Three conserved domains located at the C‐terminal of HMGA2 could bind the AT repeated sequence of DNA, which were named as “AT hook”.^35^ Each AT hook of HMGA2 contains five to six positive charges; therefore, HMGA2 should force the DNA locus to bend in a certain angle by electric repulsion,^36^ which finally induces a chromatin conformational change. Our present study demonstrated that the AT‐rich sequences of the TSS site and two conserved regions are essential for the HMGA2‐induced MMP2 transcription upregulation. Furthermore, we first confirmed that the transcriptional activation of MMP2 could be mediated by the induction of a long‐range chromatin interaction among certain cis‐regulatory elements by HMGA2.

Structural and biochemical analysis of the AT‐rich sequences only suggested the potential of HMGA2 to induce a long‐range chromatin interaction; however, other epigenetic factors may also participate in the process. Previous results showed that HMGA2 may regulate the expression of histone acetyltransferases (Hats) in pancreatic ductal adenocarcinoma.^26^ Our results showed that HMGA2 interacts with GCN5, the first identified histone lysine acetyltransferase, and recruits GCN5 to certain regions of the MMP2 promoter. The association of GCN5 with the other subunits modulates GCN5 activity and specificity.^37^, ^38^ By partnering with HMGA2, GCN5 localizes to the HMGA2 binding sites of MMP2 and catalyzes the histone H3K9 and H3H12 acetylations, finally inducing the long‐range chromatin conformational change and MMP2 upregulation.

The overexpression of MMP2 has been found in many malignant tumors.^21^, ^23^, ^39^ In our FFPE samples of grade I‐IV gliomas, the MMP2 level increased with an increase in the WHO grade, suggesting that the MMP2 level is also a potential biomarker to distinguish glioma grade. The levels of the mRNA and protein of *MMP2* were positively correlated with those of *HMGA2*, further confirming that HMGA2 overexpression promotes the cell invasion of malignant gliomas by directly activating MMP2 expression. Moreover, the tumor subgroups in the patients from our collection and those from the TCGA GBM dataset with a higher MMP2 level also had shorter DFS and OS, indicating that the MMP2 level was a serviceable biomarker for prognosis‐based glioma subclassification. Our analysis results showed that MMP2 could only predict the patient prognosis auxiliarily, and it needs other factors to precisely assess patient outcome. For example, our previous study confirmed that MMP2 is required for the MMP16‐mediated migratory and invasive phenotype of GBM.^25^ In the present study, our results determined that GCN5 knockdown significantly attenuated the promoting effects of HMGA2 on GBM cell migration and invasion, which further verified that the HMGA2/GCN5 heterodimer interaction promoted malignant glioma invasion through direct activation of *MMP2* gene expression (Figure 7H).

In summary, we revealed previously unknown mechanisms of gliomagenesis and malignant progression driven by HMGA2 via direct activation of the transcription of the *MMP2* gene, the HMGA2/GCN5 epigenetic regulation axis may provide novel targeting site in malignant glioma therapy. Moreover, we determined that HMGA2 could predict the poor prognosis of glioma patients and that HMGA2 might act as novel subclassification diagnosis factor.

## CONFLICT OF INTEREST

No conflict of interests were declared.
