# Supplementary figures and images for: HMGA2 promotes glioma invasion and poor prognosis via a long‐range chromatin interaction
Source: Cancer Med. 2018 May 7;7(7):3226–39. doi: 10.1002/cam4.1534 (PMC6051173; doi:10.1002/cam4.1534)

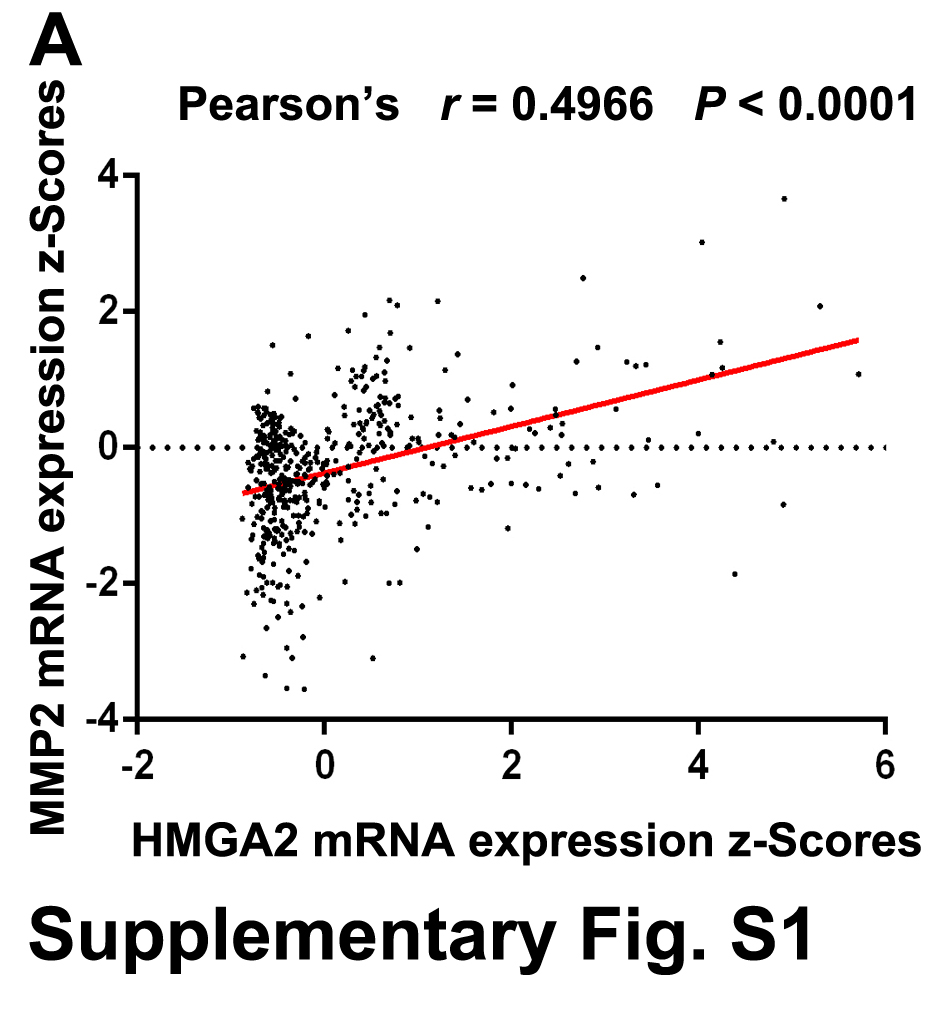

Supplement: Supplementary file 1 [file CAM4-7-3226-s001.tif]
