# Supplementary material for: HMGA2 promotes glioma invasion and poor prognosis via a long‐range chromatin interaction
Source: Cancer Med. 2018 May 7;7(7):3226–39. doi: 10.1002/cam4.1534 (PMC6051173; doi:10.1002/cam4.1534)
Supplement: Supplementary file 2 [file CAM4-7-3226-s002.doc]

**Supplementary Tables**

**Supplementary Table S1.** The clinical features of the glioma specimens used in this study

| Feature | | WHO Grade | | | |
| --- | --- | --- | --- | --- | --- |
|  |  | I (n=15) | II (n=28) | III (n=34) | IV (n=70) |
| IDH status | | | | | |
| Mutant type (IDH1 R132H) | | 0 | 25 | 28 | 3 |
| Wild type (IDH1/2) | | 15 | 3 | 6 | 67 |
| Gender |  |  |  |  |  |
|  | Male | 7 | 16 | 18 | 46 |
|  | Female | 8 | 12 | 16 | 24 |
| Age (Year, Mean ± SD) | | 13±6.9 | 41±12.2 | 46±14.7 | 60±12.1 |
| KPS | |  |  |  |  |
|  | KPS < 90 | 4 | 15 | 18 | 47 |
|  | KPS ≥ 90 | 11 | 13 | 16 | 23 |
| Predominant side | | | | | |
|  | Left | 6 | 13 | 15 | 38 |
|  | Right | 5 | 13 | 17 | 31 |
|  | Middle | 4 | 2 | 2 | 1 |
| Predominant location | | | | | |
|  | Frontal lobe | 0 | 23 | 23 | 32 |
|  | Temporal lobe | 0 | 2 | 5 | 26 |
|  | Parietal lobe | 0 | 1 | 4 | 5 |
|  | Occipital lobe | 0 | 0 | 1 | 5 |
|  | Pineal body | 1 | 0 | 0 | 0 |
|  | Saddle area | 1 | 0 | 0 | 0 |
|  | Thalamus | 1 | 0 | 0 | 0 |
|  | Cerebellum | 12 | 2 | 0 | 2 |
|  | Third ventricle | 0 | 0 | 1 | 0 |

Abbreviation: SD, standard deviation.

**Supplementary Table S2.** Primers used for promoter DNA ChIP-qPCR detection

| Primers |  | Sequence |
| --- | --- | --- |
| MMP2-promoter-1 | forward | 5’-CACGGAGACAGGAAGTGAGC-3’ |
|  | reverse | 5’-AGGTTTGTGACTACCCTGCG-3’ |
| MMP2-promoter-2 | forward | 5’-CTTCTGCAGCCATTGCCATC-3’ |
|  | reverse | 5’-GGGCAGCTTTGACTGTACCT-3’ |
| MMP2-promoter-3 | forward | 5’-TTTGCAGCCCCTGCTTTAGT-3’ |
|  | reverse | 5’-GCCAAGTTGGAGAGCTGAGA-3’ |
| MMP2-promoter-4 | forward | 5’-TCGCACTATACGAGGCCAAG-3’ |
|  | reverse | 5’-CAGTGGAAGGTCCCAGGTTG-3’ |
| MMP2-promoter-5 | forward | 5’- CCGCTGCTCTCTAACCTCAG-3’ |
|  | reverse | 5’- CCTGCTACTCCTGGCCTCTA-3’ |

**Supplementary Table S3. shRNAs and scramble used for RNA interference**

| shRNAs |  | Sequence |
| --- | --- | --- |
| HMGA2 shRNA-1 | sense | 5’- GGCCACAACAAGTTGTTCAGAA-3’ |
|  | anti-sense | 5’- TTCTGAACAACTTGTTGTGGC-3’ |
| HMGA2 shRNA-2 | sense | 5’- GAGTCCCTCTAAAGCAGCTCAA-3’ |
|  | anti-sense | 5’- TTGAGCTGCTTTAGAGGGACTTTT-3’ |
| GCN5 shRNA-1 | sense | 5’- GCTGAACTTTGTGCAGTACAA-3’ |
|  | anti-sense | 5’- TTGTACTGCACAAAGTTCAGC-3’ |
| GCN5 shRNA-2 | sense | 5’- CCACCTGAAGGAGTATCACATC-3’ |
|  | anti-sense | 5’- GATGTGATACTCCTTCAGGTGGTT-3’ |
| Scramble | sense | 5’-TTCTCCGAACGTGTCACGTTT-3’ |
|  | anti-sense | 5’-ACGTGACACGTTCGGAGAATT-3’ |

**Supplementary Table S4.** Probe used for EMSA

|  | Sequence |
| --- | --- |
| forward | 5’-ACGTTGCCATGGACGTTGCCATGGACATTGCCATGGACATTGCCATGG-3’ |
| reverse | 5’-CCATGGTAATGTCCATGGTAATGTCCATGGTAACGTCCATGGTAACGT-3’ |

**Supplementary Table S5. Primers used for mRNA qRT-PCR detection**

| Primers |  | Sequence |
| --- | --- | --- |
| HMGA2 | forward | 5’- TCCCTCTAAAGCAGCTCAAAA-3’ |
|  | reverse | 5’- ACTTGTTGTGGCCATTTCCT-3’ |
| GCN5 | forward | 5’- ACTAGTCTGGGGATGGCAGA-3’ |
|  | reverse | 5’- ATTGGAGAGTTTGCCCCATA-3’ |
| MMP2 | forward | 5’-CCCCAAAACGGACAAAGAG-3’ |
|  | reverse | 5’-CTTCAGCACAAACAGGTTGC-3’ |
| GAPDH | forward | 5’-TGCACCACCAACTGCTTAGC-3’ |
|  | reverse | 5’-GGCATGGACTGTGGTCATGAG-3’ |

**Supplementary Table S6. Primers used for mRNA 3C**

| Primers | Sequence |
| --- | --- |
| 3C-1 | 5’- TCACCAAGATGCCTGGATCTGCCTT-3’ |
| 3C-2 | 5’- GCTGGGCACAGAAGCCTCTCTCTCA-3’ |
| 3C-3 | 5’-GAGGACTGTGGCAAACCAAAGT-3’ |
| 3C-4 | 5’-AATTCGTGGAACTGAGGGCTC-3’ |
| 3C-5 | 5’-TCTGTCCCTATCCCTAAATATCG-3’ |
